# Supplementary material for: The Effect of Food Vouchers and an Educational Intervention on Promoting Healthy Eating in Vulnerable Families: A Pilot Study
Source: Nutrients. 2022 Nov 23;14(23):4980. doi: 10.3390/nu14234980 (PMC9739317; doi:10.3390/nu14234980)
Supplement: Supplementary file 1 [file nutrients-14-04980-s001.zip › nutrients-2052414-supplementary-done.pdf]

nutrients MDPI

Supplementary Materials

Table S1. Mean and SD values of other biochemical parameters before and after the intervention.

N=66	Pre-intervention		Post-intervention	
	Control= 32	Intervention= 34	Control= 32	Intervention= 34
	Mean (SD)	Mean (SD)	Mean (SD)	Mean (SD)
Red blood cells 106/µL	4.79 (0.46)	4.93 (0.39)	4.79 (0.45)	4.87 (0.37)
Haemoglobin (g/dl)	13.60 (1.27)	13.94 (1.18)	13.59 (1.17)	13.70 (1.21)
Haematocrit (%)	40.97 (3.88)	41.86 (3.57)	40.81 (3.52)	41.73 (1.18)
Leucocytes 103/µL	7.11 (1.35)	7.26 (2.15)	7.64 (1.89)	6.83 (2.06)
Erythrocyte Sedimentation Rate (mm)	6.61 (5.79)	6.94 (5.24)	8.86 (9.70)	6.64 (4.96)
Platelets 103/µL	294.81 (69.00)	289.00 (66.55)	292.21 (71.38)	275.52 (64.76)
BUN (mg/dL)	13.21 (4.04)	14.85 (4.18)	14.22 (5.28)	13.87 (3.66)
Creatinine (mg/dL)	0.58 (0.16)	0.62 (0.23)	0.61 (0.17)	0.65 (0.20)
Bilirubin (mg/dL)	0.55 (0.19)	0.66 (0.42)	0.60 (0.22)	0.70 (0.31)
Alkaline Phosphatase (U/L at 37°C)	147.70 (81.62)	152.67 (105.69)	145.07 (79.00)	149.94 (98.49)
